# Structural mimicry between SLA/LP and *Rickettsia* surface antigens as a driver of autoimmune hepatitis: insights from an *in silico* study

**DOI:** 10.1186/1742-4682-10-25

**Published:** 2013-04-10

**Authors:** Alessandro Paiardini, Stefano Pascarella

**Affiliations:** 1 Dipartimento di Scienze Biochimiche “A. Rossi Fanelli”, Sapienza - Università di Roma, Roma, 00185, Italy

**Keywords:** Autoimmune hepatitis, Molecular mimicry, *Rickettsia*, SLA/LP, Peptide conformation, PLP-dependent enzymes, HLA-DRB1*03:01

## Abstract

**Background:**

Autoimmune hepatitis (AIH) is a chronic, progressive liver disease, characterized by continuing hepatocellular inflammation and necrosis. A subgroup of AIH patients presents specific autoantibodies to soluble liver antigen/liver-pancreas (SLA/LP) protein, which is regarded as a highly specific diagnostic marker. Autoantigenic SLA/LP peptides are targeted by CD4^+^ T cells, and restricted by the allele HLA-DRB1*03:01, which confers disease susceptibility in Europeans and Americans. A positively charged residue at position 71 has been indicated as critical for AIH susceptibility in all of the HLA alleles identified to date. Though the exact molecular mechanisms underlying pathogenesis of AIH are not clear, molecular mimicry between SLA/LP and viral/bacterial antigens has been invoked.

**Methods:**

The immunodominant region of SLA/LP was used as query in databank searches to identify statistically significant similarities with viral/bacterial peptides. Homology modeling and docking was used to investigate the potential interaction of HLA-DRB1*03:01 with the identified peptides. By molecular mechanics means, the interactions and energy of binding at the HLA binding site was also scrutinized.

**Results:**

A statistically significant structural similarity between the immunodominant regions of SLA/LP and a region of the surface antigen PS 120 from *Rickettsia spp*. has been detected. The interaction of the SLA/LP autoepitope and the corresponding *Rickettsia* sequence with the allele HLA-DRB1*03:01 has been simulated. The obtained results predict for both peptides a similar binding mode and affinity to HLA-DRB1*03:01. A “hot spot” of interaction between HLA-DRB1*03:01 and PS 120 is located at the P4 binding pocket, and is represented by a salt bridge involving Lys at position 71 of the HLA protein, and Glu 795 of PS120 peptide.

**Conclusions:**

These findings strongly support the notion that a molecular mimicry mechanism can trigger AIH onset. CD4^+^ T cells recognizing peptides of SLA/LP could indeed cross-react with foreign *Rickettsia spp.* antigens. Finally, the same analysis suggests a molecular explanation for the importance of position 71 in conferring the susceptibility of the allele HLA-DRB1*03:01 to AIH. The lack of a positive charge at such position could prevent HLA alleles from binding the foreign peptides and triggering the molecular mimicry event.

## Background

Autoimmune hepatitis (AIH) is a chronic, progressive liver disease, characterized by continuing hepatocellular inflammation and necrosis. Autoantibodies to several antigens represent a serological feature of AIH, though most of them are not specific for the disease [1]. In contrast, autoantibodies to soluble liver antigen and to liver-pancreas (SLA/LP) have been described as disease specific, suggesting their potential involvement in the pathogenesis of AIH, at least in the subgroup of patients presenting SLA/LP autoantibodies (about 20% of AIH cases) [2,3]. Expression, cloning and absorption experiments identified a protein with homology to a putative UGA suppressor tRNA-associated protein, as the sole target antigen of SLA/LP autoantibodies [4]. This protein had been previously identified as it co-precipitated with tRNA^Sec^, when mammalian cell extracts were treated with serum from patients with AIH [5]. Subsequent *in vivo* and *in vitro* results therefore identified SLA/LP as *O*-phosphoserine (Sep)-tRNA:selenocysteine (Sec)-tRNA synthase (SepSecS, according to the Nomenclature Commission of the Human Genome Organization). SLA/LP belongs to the superfamily of pyridoxal 5^′^-phosphate (PLP) dependent enzymes of “fold type I” [6,7], sharing the same fold and high structural similarity with other members of this group [8,9], and catalyzing the last step of Sec synthesis, i.e., the conversion of Sep-tRNA^Sec^ to Sec-tRNA^Sec^[10]. By studying carboxy-terminally truncated SepSecS, Wies et al. [4] identified an immunodominant region that is specifically recognized by SLA/LP autoantibodies, and which is located between residues 450–490.

Whereas autoantibodies represent a serological feature of AIH, intrahepatic lymphocytic infiltrates are the histologic hallmark of AIH, and are regarded as the primary factor for disease pathogenesis [11]. Indeed, intrahepatic CD4^+^ T cells recognize self-antigens in the context of the alleles HLA-DRB1*03:01 and HLA-DRB1*0401, which represent the principal AIH susceptibility alleles among Europeans and Americans, and in the context of alleles HLA-DRB1*04:05 and HLA-DRB1*04:04, which are instead linked to AIH susceptibility in Japan, Argentina and Mexico [12,13]. In particular, a positively charged residue at position 71 in the context of the region 67–72 of the DR*β* polypeptide corresponding to the LLEQ[K/R]R motif, which is shared among the above mentioned HLA alleles, has been indicated as critical for AIH susceptibility [14]. Recently, HLA-DRB1*03:01 transgenic mice were immunized with SLA/LP, with the aim of identifying auto-antigenic SLA/LP peptides that are targeted by CD4^+^ T cells, and restricted by the disease susceptibility gene HLA-DRB1*03:01 [15]. Interestingly, the observation that the C-terminal region of SLA/LP spanning residues 452–465 (sequence NRLDRCLKAVRKER), which was identified as the optimal CD4^+^ T cell epitope, overlaps with the SLA/LP sequence that is recognized by antibodies of patients with AIH suggests that the C-terminal region of SLA/LP is not only targeted by humoral, but also by cellular immune responses [4,15].

To date, the exact molecular mechanisms that initiate and maintain the production of autoantibodies in AIH are not clear, though the remarkable uniformity in epitope recognition shown by SLA/LP autoantibodies suggests a common mechanism [16]. Molecular mimicry between self-antigens and antigens from infectious agents has been suggested as a mechanism for the generation of autoantibodies [17]. Therefore, it has been hypothesised that autoimmunity to the SLA/LP protein might be driven by viral/bacterial antigens, rather than by the SLA/LP antigen itself [16]. Nevertheless, previous attempts to look for evidence of cross-reactivity of the immunodominant region that is specifically recognized by SLA/LP autoantibodies with microbial antigens have been unsuccessful [16], leading to the conclusion that SLA/LP autoimmunity is autoantigen-driven, rather than being driven by molecular mimicry [18].

The present study suggests that local sequence similarity between SLA/LP and a non-homologous bacterial protein from *Rickettsia spp.* can drive autoimmunity to SLA/LP, through initial CD4^+^ T cell recognition and subsequent humoral response.

## Methods

### Sequence analysis

Residues encompassed by positions 450–495 of SLA/LP autoantigen (*O*-phosphoserine (Sep)-tRNA:selenocysteine (Sec)-tRNA synthase) from *Homo sapiens* [UniProt:Q9HD40, sequence variant AAD33963.2 according to [4]] were used as query in the non-redundant (nr) protein sequence database (GenBank CDS translations [19], PDB [20] and UniProtKB/Swiss-Prot [21]) search, by means of the BLAST server [22]. Algorithm parameters were kept at their default values except for word size, which was set to 2 to enhance search sensibility. Sequence display and alignment relied on the program Jalview [23]. Secondary structure and solvent accessibility were predicted with JPred3 [24]. Predictions of intrinsically disordered regions (IDRs) were carried out as described previously [25].

### Modeling of the interaction HLA-DRB1*03:01-peptide

The crystal structure of HLA-DRB1*03:01 [UniProt:P01912] in complex with a 15 residues fragment (87–101) of invariant chain called CLIP was obtained by PDB [PDB:1A6A; [26]]. The CLIP fragment was subsequently used as structural template to model the potential conformation of other interacting peptides. Molecular modeling relied on the program Molecular Operating Environment [27] and the PyMod tool [28]. *In silico* mutagenesis of the side-chains of the CLIP fragment was performed to obtain the initial complex between HLA-DRB1*03:01 and the target peptide. Then, after manual adjustment, energy minimization was performed on the whole system. Initially, to allow added hydrogens to adjust to the crystallographically defined environment, the position of the heavy atoms of the binary complex were fixed, and steepest descents steps of energy minimization were performed until the Root Mean Square gradient fell below the 0.05 Å default threshold. Next, while mainchain atoms were fixed, sidechains of every residue comprised in a sphere of 20 Å from the docked peptides were subjected to a gradually decreasing tethering force (from 1000 Kcal·Å^-2^ to 10 Kcal·Å^-2^) using again steepest descents, until the Root Mean Square gradient fell below the default threshold. Finally, a decreasing tethering force (until the system was totally relaxed) was applied on every atom comprised in a sphere of 10 Å from the docked peptides, using conjugated gradients, until the maximum gradient was less than 0.0001 Å. The Amber99 forcefield, a distance-dependent dielectric constant and a cut-off distance of 40 Å were used during each simulation.

An approximate estimate of the binding free energy for both complexes was computed by means of the FastContact 2.0 server [29]. The algorithm implemented in FastContact is based on a statistically determined desolvation contact potential and Coulomb electrostatics, and reports residue contact free energies that rapidly highlight the hotspots of the interaction.

Surface electrostatic calculations were performed by using the Adaptive Poisson-Boltzmann Solver (APBS) software [30].

## Results

### Sequence similarity and structure analysis

**Figure 1:**
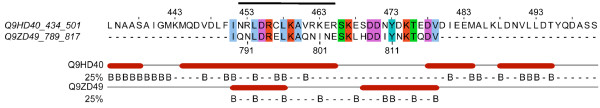
**Sequence comparison between the autoepitope of SLA/LP and the corresponding region of the 120 KDa surface antigen from *Rickettsia prowazekii.*** Sequence comparison between the region encompassing the autoepitope of the human SLA/LP antigen [UniProt:Q9HD40] and the portion of the 120 KDa surface antigen from *Rickettsia prowazekii* [UniProt:Q9ZD49]*.* Numbers attached to the accession codes indicate the sequence positions of the displayed segments within the respective entire sequences. Amino acid letters are colored according to conservation and physico-chemical properties. Annotations below alignment indicate the respective predicted secondary structure (red bars represent α-helices) and solvent accessibility. Sequence positions corresponding to B letters in the lines labeled 25% are predicted to expose to the solvent less than 25% of their total area. The black bar atop sequences denotes the peptide interacting with HLA-DRB1*03:01.

Databank searches identified a set of 120 KDa surface antigens from *Rickettsia prowazekii* (for example, PS 120 protein, UniProt:Q9ZD49), sharing a region of high (Score = 29.6 bits; Identities = 16/29 (55%); Positives = 19/29 (66%), Gaps = 0/29 (0%)) and significant (E-value = 2*e*^−04^) similarity with residues 451–479 of SLA/LP (Figure 1). The identified region of PS 120 protein spans residues 789–817 of Q9ZD49, which are evolutionarily well conserved amongst other strains and members of the *Rickettsiales*. In particular, the sequence segment DDIYNKTQDV at positions 808–817 (Figure 1) is almost invariant (see Additional file 1). Interestingly, this sequence is also conserved in SLA/LP antigen where it has been shown to be essential for immune response [16].

The observed sequence similarity between the SLA/LP immunodominant antigen and the sequence 789–817 of PS 120 kDa protein from *R. prowazekii* suggests that these polypeptide regions share a similar secondary structure context. To investigate this issue, secondary structure, accessibility and IDRs predictions were carried out on the complete sequences of SLA/LP and PS 120 protein. The results suggest the presence, for the immunodominant polypeptide region (451–490), of a helix-loop-helix secondary structure element that is accessible to the solvent. The element (helix-loop-helix) is predicted for the corresponding polypeptide region of PS 120 protein (Figure 1).

Simulation of the interaction between HLA-DRB1*03:01 and the peptides SLA/LP_452-465_, and PS 120_790-804_.

**Figure 2:**
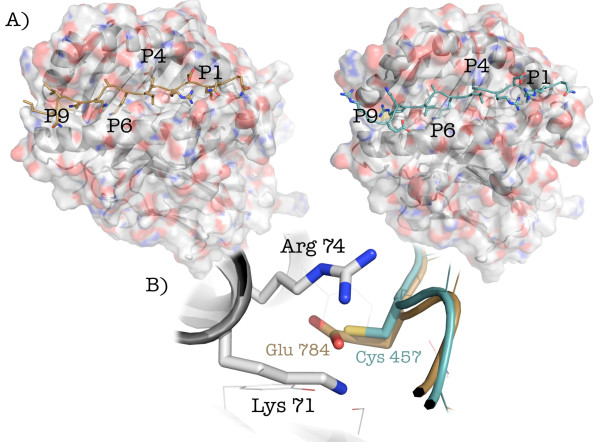
Modeling of the interaction between HLA-DRB1*03:01, SLA/LP_**452-465**_**, and PS 120**_**790-804**_**. A**) The crystal structure of HLA-DRB1*03:01 is represented as ribbons and transparent surface, with carbon atoms colored in light grey, oxygen in red, nitrogen in blue and sulfur in yellow. SLA/LP_452-465_ (peptide “A”) and PS 120_790-804_ (peptide “B”) are depicted as cyan and brown sticks, respectively. The location of anchoring pockets P1, P4, P6 and P9 is also indicated. **B**) Detail of the P4 binding cleft. This figure was rendered with PyMOL [35].

CD4^+^ T cells are the main effectors in most autoimmune diseases. Thus, molecular mimicry depends on demonstrating that these T cells can be activated by antigenic determinants of an infectious agent that are similar to the determinants present in the host [31]. A recent study on auto-antigenic SLA/LP peptides targeted by CD4^+^ T cells, pinpointed residues 452–465 (hereinafter dubbed peptide “A”, sequence NRLDRCLKAVRKER) of the C-terminal, immunodominant region of SLA/LP, as the optimal epitope for CD4^+^ T cells expressing the AIH disease susceptibility gene HLA-DRB1*03:01 [15]. As shown in Figure 1, the peptide “A” shares high sequence similarity to the sequence QNLDRELKAQNINE encompassed by positions 790–803 of the PS 120 from *Rickettsia prowazekii*, (hereinafter called peptide “B”). However, since it is well known that sequence similarity alone is not sufficient for mimicry in autoimmune diseases, we decided to model the interaction of HLA-DRB1*03:01 with peptide “A”, whose interaction has been already proven, and to compare it with a hypothetical complex between HLA-DRB1*03:01 and peptide “B” (Figure 2). Residue Leu 454 of peptide “A” (corresponding to Leu 792 of peptide “B”) was chosen as hydrophobic anchoring sites at the P1 pocket, since: 1) it is well known that filling this pocket with a hydrophobic residue constitutes a primary requirement for epitope binding to HLA and epitope selection [32,33]; 2) the P1 pocket of HLA-DRB1*03:01 is located at the beginning of the peptide binding groove, therefore the hydrophobic anchor site should be located at the N-terminus or C-terminus of an interacting peptide, and Leu 454 is the only hydrophobic residue of peptide “A” fulfilling this requirement. Following the choice of the P1 pocket anchoring site, the main-chain of peptide “A” and peptide “B” were initially modeled by assigning the coordinates of the CLIP fragment. The rationale of this procedure is that all of the peptides binding to MHC class II adopt a similar type II polyproline helix conformation, as they interact with the binding groove [34]. *In silico* mutagenesis of the side-chains of the CLIP fragment was then performed to obtain initial HLA-DRB1*03:01-peptide “A” and HLA-DRB1*03:01-peptide “B” complexes. The side-chain rotamers of peptide “A” and peptide “B” were chosen in order to keep the most similar orientation of the corresponding CLIP peptide, and to interact with the same functional residues. Particular care was taken to remove the steric clashes in the resulting complex.

After manual docking and energy minimization of the complexes, peptide “A” and “B” showed good predicted binding affinities for HLA-DRB1*03:01 (−33.6 Kcal/mol for peptide “A” and −28.1 Kcal/mol for peptide “B”). Both peptides twist in the typical type II polyproline helix, with the sequestration of peptide side chains in polymorphic P1, P4, P6, and P9 pockets in the HLA protein, which have been identified as major anchors [33]. Table 1 reports the receptor-ligand residue free energy contacts for HLA-DRB1*03:01-peptide “A” and HLA-DRB1*03:01-peptide “B” complexes. As expected, residue Leu 454 of peptide “A” (Leu 792 of peptide “B”) fits well in the highly hydrophobic P1 pocket, formed by residues Phe 24, Ile 31, Phe 32, Trp 43, Ala 52, Asn 82, Val 85, Val 86 and Phe 89 of HLA-DRB1*03:01 (predicted desolvation free energy: −5.624 Kcal/mol and −5.440 for peptide “A” and “B”, respectively). According to Table 1, a “hot spot” of interaction between HLA-DRB1*03:01 and peptide “B” is located at the P4 binding pocket, and is represented by the salt bridges involving Lys at position 71 and Arg at position 74 of the HLA protein, and Glu 795 of PS120 peptide (Lys 71-Glu 795, −5.191 Kcal/mol; Arg 74-Glu 795, −3.153 Kcal/mol). These interactions are not present in SLA/LP immunodominant peptide, where the Glu residue is substituted for Cys 457. The P6 binding pocket of HLA-DRB1*03:01 is negatively charged, being occupied by residues Glu 9 of chain A, Glu 11 and Asp 66 of chain B. In this pocket are accommodated the positively charged residue Lys 459 and Lys 797 of peptides “A” and “B”, respectively, which favourably contribute to the free energy of binding (−3.609 Kcal/mol and −4.072 Kcal/mol, respectively). Finally, the positively charged side-chain of Arg 462 of peptide “A” is located inside the P9 binding pocket, where it interacts with Asp 57 (chain B) of HLA-DRB1*03:01. The latter represents the most favourable interaction between HLA-DRB1*03:01 and peptide “A”, according to residue free energy contacts (−7.382 Kcal/mol). In case of peptide “B”, Arg 462 is replaced by Asn 800, which is hydrogen-bonded to Asn 69 (chain A) and Asp 57 (chain B) of HLA-DRB1*03:01.

**Table 1:** Top 10 hot spots of interaction (according to free energy of binding) between HLA-DRB1*03:01, human SLA/LP_**452-465**_**, and the corresponding PS 120**_**790-804 **_**from *R. prowazekii***

HLA-DRB1*03:01	**SLA/LP**	**Free energy (Kcal/mol)**
Asp B57	Arg 462	−7.382
Glu A55	Arg 453	−5.015
Asp A66	Lys 459	−4.108
Glu B9	Lys 549	−2.518
Arg A76	Arg 465	−2.381
Leu B53	Arg 462	−2.013
Ser B11	Lys 459	−1.879
Tyr B78	Cys 457	−1.158
Val B85	Leu 454	−1.130
Phe A54	Leu 454	−1.081
HLA-DRB1*03:01	**PS 120**	**Free energy (Kcal/mol)**
Lys B71	Glu 795	−5.191
Asp A66	Lys 797	−3.279
Arg B74	Glu 795	−3.153
Glu B9	Lys 797	−2.671
Arg B74	Leu 796	−1.972
Asp B57	Asn 800	−1.425
Tyr B61	Ile 801	−1.235
Glu A55	Arg 794	−1.211
Phe A54	Leu 792	−1.121
Val B85	Leu 792	−1.057

## Discussion

PLP-enzymes are involved in a number of diseases, including autoimmunity [36-38], and Bioinformatics approaches have been extensively exploited in the past to understand the molecular basis of human disorders involving PLP-enzymes [39,40], and to design drugs specifically targeting such enzymes [41,42].

This study suggests the possible role of molecular mimicry between microbial antigens and the immunodominant region of a PLP dependent enzyme, SLA/LP. The hypothesis that foreign antigens of bacterial proteins sharing homology with the SLA/LP protein might drive autoimmunity, via a molecular mimicry mechanism, has been already debated [16]. By performing sequence similarity searches in publicly available databases, these authors identified the protein MJ0610 from the Archaea *Methanococcus jannaschii* [GenBank:U67509], a non-pathogenic, hyperthermophillic organism, as the only bacterial candidate for molecular mimicry to occur. Subsequent attempts to look for evidence of cross-reactivity of the immunodominant region that is specifically recognized by SLA/LP autoantibodies with MJ0610 have been unsuccessful [16]. The huge amount of sequence information that is available nowadays in protein databases prompted us to search again for bacterial proteins that could be able to trigger an autoimmune response in AIH, on the base of a molecular mimicry event. The obtained results suggest that a highly significant, local sequence similarity between SLA/LP and a non-homologous bacterial protein from *Rickettsia spp.* might drive autoimmunity to SLA/LP, through initial CD4^+^ T cell recognition and subsequent humoral response.

The PS 120 kDa protein from *R. prowazekii* is a 1022 residues sequence of unknown function, belonging to the family of 120 KDa Rickettsia surface antigens [Pfam: 12574] [43], which may be used as antigens for immune response against the *Rickettsia* species [44]. This protein is a close homolog of *R. conorii* PS 120 kDa protein [UniProt:Q52658; identities = 640/1019 (63%)], an antigen that is recognized by antirickettsial antibodies in sera from humans infected with spotted fever group rickettsiae, and that is supposed to be an important stimulator of the host immune response [44].

Together with mono-dimensional amino acid sequence similarity, the three-dimensional conformational fit of the immunodominant epitope from host with a polypeptide chain from the pathogen is also a key aspect in for molecular mimicry to occur [45]. Since SLA/LP autoantibodies react preferentially with conformational epitopes [46], we considered the possibility that, beside sequence similarity, the SLA/LP immunodominant antigen and residues 789–817 of PS 120 kDa protein from *Ricketsia spp.* could present a similar secondary structure motif and solvent accessibility. The crystal structure of the human SepSecS-tRNA^Sec^ binary complex [PDB: 3HL2] revealed that the first 14 residues (450–463) of the immunodominant region of SLA/LP adopt an α-helix secondary structure, while the remaining residues (464–501) showed a disordered state and were not solved in any of the crystal structures of human SepSecS already determined [47-49]. Our structural analysis on the immunodominant region of SLA/LP agrees with previous bioinformatics studies [50], suggesting that residues 450–490 of SLA/LP fold in a helix-loop-helix conformation that is accessible to the solvent, and therefore easily recognized by SLA/LP autoantibodies. Moreover, we suggest that the corresponding region of PS 120 KDa protein could adopt a similar conformation and solvent exposure, which therefore would render it amenable to the proteolytic cleavage that is necessary for the immune response to take place.

The recent findings that, of two immunodominant T cell peptides of the SLA/LP protein, one overlaps with the immunodominant region that is recognized by SLA/LP autoantibodies [15], provide an interesting link between humoral and cell-mediated immune response in AIH, and prompted us to investigate further this issue, by modeling the interaction between the AIH susceptibility allele HLA-DRB1*03:01, human SLA/LP_452-465_, and the corresponding PS 120_790-802_ from *R. prowazekii*. The obtained complexes predict for both peptides a similar binding mode and affinity, suggesting that CD4^+^ T cells recognizing self-antigens in the context of the alleles HLA-DRB1*03:01 could indeed cross-react with foreign *Rickettsia spp.* peptides. Most importantly, a “hot spot” of interaction between HLA-DRB1*03:01 and peptide “B” is represented by the salt bridges involving Lys at position 71 and Arg at position 74 of the HLA protein, and Glu 795 of PS120 peptide. This observation could potentially provide a mechanistic explanation to the fact that a positively charged residue at position 71 has been indicated as critical for AIH susceptibility in all of the HLA alleles identified to date [14]. Indeed, the Lys 71-Glu 795 interaction could be essential for HLA-antigen recognition and binding, and the lack of this interaction could prevent HLA alleles from binding the foreign peptides and triggering the molecular mimicry event. Indeed, in the DRB1*1501 allele, which confers resistance to AIH [51], Lys 71 is replaced by an Ala residue. Moreover, beside Lys 71, the presence of Arg at position 74 is also related to an increased susceptibility to AIH [51]. Interestingly, in type 1 diabetes mellitus, where molecular mimicry has been proposed as an explanation of the pathogenesis of the disease, a similar mechanism has been observed: an aspartate residue at position 57 of the DQb-β polypeptide chain confers protection, whereas a serine is associated with an increased risk of disease [52].

*Rickettsia* is a genus of Gram-negative, obligate intracellular parasites, which are carried by many ticks, fleas, and lice, and cause diseases in humans such as typhus, rickettsialpox, Boutonneuse fever, African tick bite fever, Rocky Mountain spotted fever, Flinders Island spotted fever and Queensland tick typhus (Australian Tick Typhus) [53]. Actually, rickettsial microorganisms are not necessarily pathogenic species, and are indeed ubiquitous in human populations, where they normally live in peaceful coexistence with the human beings [54]. These organisms represent one of the closest living relatives to bacteria that originated the mitochondria inside most eukaryotic cells [55]. Rickettsiales display small, degraded genomes, with a high propensity of genetic exchange occurring between bacteria that infect the same host and with the eukaryotic hosts themselves. Crosstalk between host and bacteria appears to be mediated by proteins containing motifs with high similarity to eukaryotic-like repeats [53]. The widespread presence of rickettsial microorganisms amongst humans, the bacteria-host genetic exchange and the local similarity of eukaryotic-like repeats, led in the past to the hypothesis that rickettsiales are indeed related to the emergence of human autoimmune diseases, e.g., multiple sclerosis [54].

## Conclusions

Diagnosis of AIH is usually based on a number of outcomes from clinical, laboratory, and histological exams. Anti-SLA/LP autoantibodies are considered highly specific markers of AIH [2], and SLA/LP autoantibodies recognize the SLA/LP antigen with high sensitivity and specificity [4]. On the contrary, commonly detected autoantibodies such as antinuclear and smooth muscle antibodies are not specific for the disease [3,4]. The presence of different autoantibodies, that is definitively an important part of the final diagnosis, reflects the complex and diverse interplay between environmental triggering factors, autoantigens and immunogenetic predisposition of the individual.

Though it is highly unlikely that any single infectious agent would be exclusively associated with the disease, molecular mimicry provides an elegant framework as to how cross-reactivity between antigens from a foreign agent with self-proteins may trigger such disease.

The predicted sequence and structural similarity between the immunodominant epitopes of the SLA/LP antigen and PS 120 protein from *Rickettsia spp.* could account for cross-recognition to occur in autoimmune hepatitis, and contribute to the development of this disease. The presented findings are supported by coherent and rigorous theoretical considerations and form the basis of a well-grounded hypothesis that can be experimentally tested. Obviously, CD4^+^ T cell recognition requires that antigen is degraded by pH-dependent proteolysis in phago-endosomal compartments of APCs. This means that only a portion of peptides are efficiently generated from a given antigen and result available to be loaded onto MHC class II molecules. Several cryptic peptides might not have *in vivo* relevance, despite being potential MHC class II binders, if they are not generated during proteolysis of the entire protein.

To conclude that the suggested peptides are authentic immunodominant T-cell epitopes, future studies should be aimed at detecting also the frequency of memory B and T cells specific to PS 120 protein epitope in AIH patients and healthy donors. If such studies will be confirmed, they may contribute to open new perspectives for AIH prevention and therapy.

## Competing interests

The authors declare that they have no competing interests.

## Authors’ contributions

AP carried out structural analysis, helped in sequence analysis and participated in drafting the manuscript. SP carried out sequence analysis, conceived the study and participated in drafting the manuscript. Both authors read and approved the final manuscript.

## Supplementary Material

Additional file 1: **Sequence alignment of PS 120 protein (region 789–817) from different *Rickettsiales*.** Numbering refers to *R. prowazekii*. Click here for file
