# Supplementary material for: Structural mimicry between SLA/LP and Rickettsia surface antigens as a driver of autoimmune hepatitis: insights from an in silico study
Source: Theor Biol Med Model. 2013 Apr 10;10:25. doi: 10.1186/1742-4682-10-25 (PMC3636016; doi:10.1186/1742-4682-10-25)
Supplement: Additional file 1: — Sequence alignment of PS 120 protein (region 789–817) from different Rickettsiales. Numbering refers to R. prowazekii. [file 1742-4682-10-25-S1.pdf]

Supplementary Figure 1

```
                         790       800       810
                           .         .         .
R.prowazekii            IQNLDRELKAQNINESKLHDDIYNKTQDV
R.typhi                 IQNLDGELKEKNIEESKLRDDIYNKTQDV
R.akari                 TQNFTEELKGQNLDEPKPRDDIYNKAQDI
R.amblyommii            TQNLTEGLKGQNIEEHKPRDDIYNKAQEV
R.heilongjiangensis     TQNLTEGLKGQNIEEHKPHDDIYNKVREV
R.japonica              TQNLTEGLKGQNIEEHKPHDDIYNKVREV
R.sibirica              TQNLTEGLKGQNIEEHKPHDDIYNKAREV
R.philipii              TQNLTEGLKGQNIEEHKPHDDIYNKAREV
R.rickettsii            TQNLTEGLKGQNIEEHKPHDDIYNKAREV
R.peacockii             TQNLTEGLKGQNIEEHKPHDDIYNKAREV
```

Sequence alignment of PS 120 protein (region 789-817, numbering refers to *R. prowazekii*) from different *Rickettsiales*.
